# Quality of life in older adults with mood states associated with bipolar disorder: A secondary analysis of the English longitudinal study of ageing data

**DOI:** 10.1111/bjc.12495

**Published:** 2024-08-11

**Authors:** Aaron Warner, Carol Holland, Fiona Lobban, Lee Bentley, Elizabeth Tyler, Jasper Palmier‐Claus

**Affiliations:** ^1^ Division of Health Research, Spectrum Centre for Mental Health Research Lancaster University Lancaster UK; ^2^ Division of Health Research, Centre for Ageing Research Lancaster University Lancaster UK; ^3^ Lancashire and South Cumbria Care NHS Foundation Trust Lancashire UK; ^4^ Manchester Metropolitan University Manchester UK; ^5^ Division of Psychology and Mental Health University of Manchester Manchester UK

**Keywords:** ageing, bipolar, ELSA, mania, quality of life

## Abstract

**Objectives:**

To investigate: (i) whether mood states associated with bipolar disorder are associated with poorer quality of life in older adults, and (ii) what are some of the predictors of quality of life in older adults with mood states associated with bipolar disorder.

**Methods:**

The authors completed a cross‐sectional multilevel analysis of panel data from seven waves of The English Longitudinal Study of Ageing dataset. The main analysis included 567 participants who reported experiencing mood states associated with bipolar disorder. Some participants reported this in more than one wave, resulting in 835 observations of mood states associated with bipolar disorder across the seven waves. Quality of life was assessed using the Control, Autonomy, Self‐realization, and Pleasure‐19 (CASP‐19) measure.

**Results:**

The presence of mood states associated with bipolar disorder was significantly associated with poorer quality of life, even after controlling for multiple covariates (age, sex, social isolation, loneliness, alcohol use, education level, and economic status). Loneliness significantly predicted poorer quality of life in older adults with mood states associated with bipolar disorder. In contrast, higher educational attainment and being female predicted better quality of life in this group.

**Conclusions:**

Older adults with mood states associated with bipolar disorder have potentially worse quality of life compared to the general population, which may be partly driven by loneliness. This has ramifications for the support offered to this population and suggests that treatments should focus on reducing loneliness to improve outcomes.

**Practitioner points**

- Ageing with mood states associated with bipolar disorder potentially results in poorer quality of life compared to the general ageing population.
- For people ageing with mood states associated with bipolar disorder, loneliness appears to be an important predictor or poorer quality of life.
- Increased support is needed for older adults with mood states associated with bipolar disorder to reduce the challenges they experience and improve their quality of life.

## INTRODUCTION

The World Health Organization (2023) defines quality of life as ‘an individual's perception of their position in life in the context of the culture and value systems in which they live and in relation to their goals, expectations, standards and concerns’. Quality of life encompasses physical, occupational, social, and spiritual wellbeing, and can fluctuate over time due to people's changing circumstances, making it challenging to measure accurately (Michalak et al., 2005). Michalak et al. (2006) suggest that quality of life may be a key indicator of people's wellbeing and is important to consider when completing health‐related research. Assessing quality of life can enable researchers to identify the main difficulties that lead to diminished wellbeing in marginalized populations, such as older adults and those experiencing mental health difficulties (Sachs & Rush, 2003; Şahin et al., 2019). Improving our understanding of what affects quality of life in these groups can help us to consider necessary adaptations to care that can enhance the support offered (Depp et al., 2006).

A wealth of research highlights that old age can bring several challenges and losses that potentially contribute to decreased quality of life (Baernholdt et al., 2012). For example, Hawton et al. (2011) suggest that social isolation caused by reduced social contact in later life has a profound impact on quality of life as people age. Indeed, 11–17% of people aged 65 and over in the United Kingdom report feeling socially isolated and 13% of older adults living alone feel detached from society (Barnes et al., 2006). A longitudinal study by Victor and Bowling (2012) reported that changes in social networks, living arrangements, and poor physical health resulted in 9% of older adults feeling severely lonely and a further 30% feeling lonely at times (Victor & Bowling, 2012). Older age is also commonly associated with reduced physical functioning, and this has been found to be a predictor of the onset and persistence of mental health difficulties in older adults (Geerlings et al., 2000).

Living with mental health difficulties such as bipolar disorder can drastically affect people's wellbeing (IsHak et al., 2012). Bipolar disorder is defined as a chronic mood disorder characterized by extreme fluctuations in mood that significantly impact functioning (Carvalho et al., 2020). It is present in approximately 1% of the population worldwide making it one of the leading causes of disability among adult populations (Grande et al., 2016). Due to the significant mental and physical health challenges associated with bipolar disorder, many people report that living with these experiences has a profoundly negative impact on their quality of life (Michalak et al., 2006). For example, this diagnosis has been linked to reduced social support, increased stigma, increased physical health comorbidities, poorer socioeconomic status, and higher levels of substance misuse, which all lead to poorer wellbeing, marginalization, and worse clinical outcomes compared to the general population (Carvalho et al., 2020; Gutiérrez‐Rojas et al., 2008; Hawke et al., 2013). There is also evidence that people without a formal diagnosis, but experiencing extreme mood states (e.g., mania), are at risk of diminished functioning that is difficult to treat effectively (Evans et al., 1995).

Older adults experiencing bipolar disorder reportedly face both general difficulties associated with ageing and disorder‐specific difficulties (Depp & Jeste, 2004). This group have been found to experience increased physical health comorbidity, worse cognitive function, and accelerated cognitive decline compared to the general ageing population (Gildengers et al., 2009; Schouws et al., 2016; Warner et al., 2023). Consequently, there is potentially an important interaction effect between ageing and bipolar disorder that leads to poorer outcomes (Sajatovic et al., 2015).

To date, there is little research exploring quality of life outcomes in older adults with bipolar disorder. Depp et al. (2006) completed a study reporting that older adults with bipolar disorder (*n* = 54) experienced worse functioning, and poorer wellbeing in comparison to non‐clinical controls (*n* = 38), even when they were deemed to be in remission. These results did not change after controlling for covariates such as education, age, and occupational attainment. Further, large‐scale research using representative samples from the general population is needed to better understand whether older adults with bipolar disorder experience lower quality of life. Knowledge of the factors contributing to poor quality of life would also help to identify important targets for intervention in older adults experiencing mood states associated with bipolar disorder.

This study analyses data from The English Longitudinal Study of Ageing (ELSA), a large epidemiological dataset of older adults in England, to examine whether mood states associated with bipolar disorder are associated with quality of life in older adults. The term ‘mood states associated with bipolar disorder’ has been used throughout as the authors combined results for participants reporting experiences of bipolar disorder or mood swings. Consequently, whilst these experiences appear to be associated with symptoms of bipolar disorder, it is not possible to determine if participants had received a formal diagnosis of bipolar disorder in their lives. The study also investigates predictors of quality of life (loneliness, social isolation, socioeconomic status, education level, alcohol use, age, and sex) in older adults experiencing mood states associated with bipolar disorder. These predictors were selected based on existing literature surrounding older adults and individuals with bipolar disorder. For example, people with bipolar disorder are reported to experience increased loneliness, social isolation, and increased alcohol use compared to the general population (Farren et al., 2012; Lee et al., 2022; Nilsson, 2016), whilst a link between socioeconomic status and quality of life has been found in research exploring older adults with mental health difficulties (Achdut & Sarid, 2020). Consequently, these factors potentially impact quality of life in older adults with mood states associated with bipolar disorder and this will be examined within this study.

The hypotheses are:

1. The presence of mood states associated with bipolar disorder will predict poorer quality of life in an older adult sample.
2. Demographic and lifestyle factors (loneliness, social isolation, economic status, education level, alcohol use, age, and sex) will predict quality of life in older adults with mood states associated with bipolar disorder.

## METHODS

### Design

The authors completed a cross‐sectional multilevel analysis on panel data from across seven waves of The English Longitudinal Study of Ageing data (ELSA; Steptoe et al., 2013). Two waves (1 and 2) were omitted from the analysis as they did not record data for the variables being assessed within this study.

### Sample

The ELSA is an ongoing prospective observational study of community‐dwelling individuals in England, aged 50 and over (Steptoe et al., 2013). The original sample was taken from households that had taken part in the Health Survey for England (HSE) between 1998 and 2001. The main fieldwork for ELSA began in 2002 and the same group of respondents have completed two‐yearly interviews, known as waves, to assess key factors related to ageing. For this study, the researchers combined data from waves 3–9 (2006–2019), based on the availability of key variables to examine quality of life in older adults with mood states associated with bipolar disorder.

In total, 54,565 observations across 14,819 participants from seven waves of ELSA data were included in the analysis (Table 1). Within this, there were 567 participants who reported experiencing mood states associated with bipolar disorder. Some of these individuals reported this in more than one wave, resulting in 835 observations of mood states associated with bipolar disorder across the seven waves. The average number of observations for each participant was 3.6 (range 1–7). The mean age of participants with mood states associated with bipolar disorder was 62.4 years old compared to 66.8 years old for participants who did not experience mood states associated with bipolar disorder. The majority of participants were female, for both those who experienced mood states associated with bipolar disorder (54.9%) and those who did not (55.0%), indicating no clear difference in gender distribution between the two groups.

**TABLE 1:** Demographic and clinical information.

Characteristics	Mood states associated with bipolar disorder (*n* = 567 participants)	No mood states associated with bipolar disorder (*n* = 14,252 participants)
Mean age, years (range; SD)	62.4 (50–89; 9.04)	66.8 (50–90; 10.15)
Gender, *n*		
Male	256 (45.1%)	6412 (45.0%)
Female	311 (54.9%)	7844 (55.0%)
CASP‐19, mean (range, SD)	33.5 (0–53, 6.72)	37.1 (0–57, 4.72)
Loneliness, mean (range, SD)	5.56 (3–9, 2.03)	4.08 (3–9, 1.40)
Social isolation, mean (range, SD)	2.50 (0–5, 1.18)	2.34 (0–5, 1.05)
Education level, *n* (%)		
No qualifications	150 (28.7%)	3187 (24.1%)
Intermediate qualifications	292 (55.9%)	7604 (57.5%)
Higher education	80 (15.3%)	2437 (18.4%)
Alcohol use, *n* (%)		
Does not drink	89 (17.1%)	2512 (18.7%)
Irregular drinker	159 (30.6%)	4332 (32.2%)
Regular drinker	271 (52.3%)	6602 (49.1%)
Total (non‐pension) net wealth, mean £	£226,783	£383,330

### Variables

#### Dependent variable

##### Control, Autonomy, Self‐realization, and Pleasure‐19 (CASP‐19; Hyde et al., 2003)

The ELSA includes the CASP‐19 as a measure of quality of life. The CASP‐19 consists of 19 items covering four domains that were initially developed to assess quality of life in old age: control, autonomy, self‐realization, and pleasure (Wiggins et al., 2004). The control domain comprises of four items, whereas the autonomy, self‐realization, and pleasure domains all consist of five items. Each item included in this measure was scored on a four‐point Likert scale that ranged from 0 to 3 with responses ranging from ‘this applies to me: never; not often; sometimes; often’. Scores ranged from 0 to 57 with higher scores indicating better quality of life. A study completed by Hyde et al. (2003) found the CASP‐19 to be a useful, valid, and reliable scale for measuring quality of life in older adults. The authors reported that all domains on the CASP‐19 have Cronbach's alphas between .6 and .8 and concurrent validity was strong (*r* = .63, *p* = .010) when assessed alongside the Life Satisfaction Index (Liang, 1984).

#### Independent variable

##### Mood states associated with bipolar disorder

The authors identified individuals who had experienced mood states associated with bipolar disorder at any point in their lives based on participants' responses to the following question: What type of emotional, nervous, or psychiatric problems [^do (did) you /does (did) [^name]]|have? There were then the options: ‘mood swings’ and ‘manic depression’. Endorsement of either item was coded as a scoring position for mood states associated with bipolar disorder. Due to the low frequency of participants scoring positive for manic depression/bipolar disorder (*n* = 100) and mood swings (*n* = 577) across the seven waves, the authors decided to combine the variables to ensure adequate power for the analyses. This decision was deemed sensible through consultation with public advisors with bipolar disorder.

#### Other variables/covariates

##### Loneliness

Loneliness was assessed using the short form of the University of California, Los Angeles (UCLA) Loneliness scale (Russell et al., 1978). This measure has been frequently used in studies assessing loneliness using ELSA data (Bu et al., 2020; Victor & Pikhartova, 2020) and was found to have an acceptable level of internal consistency (*α* = .84) and satisfactory psychometric properties (Neto, 2014; Shankar et al., 2011). The measure contains items that ask participants to respond to how often they felt ‘isolated from others’, how often ‘they felt lonely’, and how often they ‘felt left out. Responses to these items were rated from 1 to 3 and then summed to produce a loneliness score that ranged from 3 to 9, with higher scores representing higher levels of loneliness among participants.

##### Social isolation

Social isolation was measured using the validated index developed by Shankar et al. (2011). This index was based on participants being given a score of 1 for each of the following: having less than monthly contact (including on the telephone, face‐to‐face, using email, or text messaging) with family members, friends, or children. Participants were also given a score of 1 for not belonging to any clubs or social organizations and for living alone. Scores ranged from 0 to 5 with higher scores indicating increased isolation among participants in the study.

##### Economic status

As recommended by Shankar et al. (2010), total non‐pension net wealth was used to measure economic status. Total non‐pension net wealth estimated the value of participants' financial assets minus any debt. This variable was split into quartiles (quartile one: lowest economic status; fourth quartile: highest economic status).

##### Education (categorical variable)

Education was measured by classifying participants into three separate groups depending on their highest qualification achieved. Education level was collapsed into three categories as suggested by Shankar et al. (2010). Participants were categorized as having ‘no qualifications’, highlighting that the individual had left education without gaining any qualifications. The second category was ‘intermediate qualifications’, indicating that the individual had high school qualifications below degree level (e.g., o‐level, a‐level, National Vocational Qualifications (NVQ) at levels 1–3); and category three was defined as higher education, identifying participants who had degree‐level qualifications, NVQ at levels 4 or 5, or higher degrees.

##### Alcohol use (categorical variable)

The authors controlled for alcohol use with a measure detailing how frequently participants consumed alcohol over the past 12 months. This measure was categorized into three groups: Group one consisted of participants who ‘do not drink’ or had not had an alcoholic drink in the past 12 months (Holdsworth et al., 2016). Category two was defined as ‘irregular drinkers’ consisting of participants who reported drinking monthly or less (Mazzaglia et al., 2001); category three identified participants who drank alcohol above this threshold (weekly or more) and were defined as ‘regular drinkers’.

#### Other

Data was also available for the age of participants (collapsed at age 90). That is, age was recorded to a maximum of age 90 to minimize the risk of disclosure for the small number of participants aged over 90. The sex (male or female) of participants was also available. These variables were included as covariates in the analysis.

### Missing data

Missing values are a problem that is frequently encountered by researchers who analyse large datasets. Missing data can arise due to issues such as dropout or non‐responses from study participants and can lead to smaller sample sizes that can compromise the validity and reliability of findings (Kwak & Kim, 2017). A number of methods can be used to account for missing data and reduce the risks of producing biased results or incorrect inferences about the population being examined (White & Carlin, 2010). In the present study, the researchers utilized complete case analysis. This method involves only including complete data whilst excluding all cases with missing data from the analysis (Little et al., 2022). Its simplicity and efficiency present an advantage, although critics argue that this method can lead to smaller sample sizes and reduced statistical power, which can cause imprecision in the results (Little et al., 2022). However, complete case analysis is the most commonly used method of managing missing values within big data research (Ross et al., 2020) and was determined to be an appropriate method for accounting for missing data within this study. Missing data was addressed on a per‐model basis. Within the ELSA data, there was variation in response rates for different variables across different waves. This resulted in a different number of participants being included in each statistical model due to the availability of data. It is noted that in the current dataset, missingness due to the severity of bipolar disorder would underestimate the size of the effects (type II error rather than type I error).

### Statistical analysis

All analysis was completed in Stata 16.0. Multilevel modelling (‘xtreg’) was used to assess whether the presence of mood states associated with bipolar disorder (independent variable) predicted poorer quality of life (dependent variable) in older adults. Participants' unique ID number was entered as the random effect to account for the nested data structure. Multilevel modelling is a well‐established method for analysing data that includes a hierarchical structure where there are repeated measures of the same variables within individuals over time (Greenland, 2000). Consequently, this approach was appropriate for the present study where data was collected in seven waves ranging 2006–2019. A multilevel approach allowed the authors to model for random error and reduce the possibility of estimation error (Lara et al., 2014).

The analysis was run in multiple stages. In Model 1A, the authors ran an unadjusted model where the presence of mood states associated with bipolar disorder (independent variable) was entered as a predictor of quality of life (dependent variable), without any covariates. In Model 1B, the authors ran an adjusted model whereby mood states associated with bipolar disorder were entered as a predictor of quality of life whilst adjusting for key covariates (age, sex, loneliness, social isolation, education level, socioeconomic status, and alcohol use). A separate model (model 2) was used to explore predictors of quality of life in people with mood states associated with bipolar disorder only to test Hypothesis 2. The authors controlled for the wave of the study in models 1B and model 2.

Bootstrapping (10,000 reps) was used to account for parametric assumptions not being met in the data. Bootstrapping is a non‐parametric method of hypothesis testing and effect size estimation that is used to navigate problems around nonnormalities in the sample distribution by accounting for biases caused by the central tendencies of the estimate (Preacher & Hayes, 2004). The authors also tested for multicollinearity in Stata using the variance inflation factor (VIF) command to assess whether there was a linear relationship between the variables selected (Alin, 2010). All variables' VIFs were below 5 and were only moderately correlated; multicollinearity was not at a problematic level to increase the standard error of the coefficients included in the analysis according to the cut‐off recommended by Daoud (2017).

## RESULTS

### Sample characteristics and clinical information

Participants who experienced mood states associated with bipolar disorder were less likely to have completed higher education (15.3% vs. 18.4%) and were more likely to be categorized as regular drinkers (52.3% vs. 49.1%). The mood states associated with bipolar disorder group also reported a lower mean net wealth (£226,783 vs. £383,330), scored higher for loneliness (mean 5.56 vs. 4.08) and social isolation (mean 2.50 vs. 2.34), and scored lower on average for the CASP‐19 quality of life measure (mean 33.5 vs. 37.1; Table 1).

### Quality of life in older adults with mood states associated with bipolar disorder

#### Model 1A

Model 1A examined whether mood states associated with bipolar disorder predicted quality of life in older adults without covariates. This model showed that mood states associated with bipolar disorder significantly negatively predicted quality of life (*B*: −1.70; SE .21; CI −2.11 to −1.28; *p* < .001). Therefore, those who endorsed mood states associated with bipolar disorder scored on average 1.7 points lower on the CASP‐19 quality of life scales than those who did not endorse this item.

#### Model 1B

Model 1B was adjusted, and covariates were entered alongside mood states associated with bipolar disorder as predictors of quality of life in older adults. The negative effects of mood states associated with bipolar disorder remained significant (*B*: −1.37; SE .20; CI −1.78 to −.97; *p* < .001) after controlling age, sex, loneliness, social isolation, economic status, alcohol use, and education level and did not greatly reduce. Older adults reporting mood states associated with bipolar disorder scored, on average, 1.4 points lower on the quality‐of‐life scale compared to those without mood states associated with bipolar disorder.

All covariates included in the analysis were shown to be significant predictors of quality of life in the sample of older adults. Higher loneliness (*B*: −.79; SE .02; CI −.82 to −.75; *p* < .001) and social isolation (*B*: −.21; SE .02; CI −.25 to −.17; *p* < .001) were associated with poorer quality of life. Higher education level (*B*: 1.10; SE .08; CI .94–1.25; *p* < .001) and greater economic status (Quartile 4, *B*: .97; SE .09; CI .81–1.13; *p* < .001) positively predicted increased quality of life. Older age (*B*: .01; SE .02; CI .01–.02; *p* < .001), being female (*B*: .47; SE .04; CI .39–.55; *p* < .001), and being a regular drinker (*B*: .74; SE .09; CI .56–.92; *p* < .001) also resulted in positive effects in predicting quality of life in older adults (Table 2). Notably, the effects of covariates on quality of life were smaller than those for mood states associated with bipolar disorder.

**TABLE 2:** Quality of life in older adults with mood states associated with bipolar disorder.

Model	DV	IV	*B*	BS SE	*P*	CI lower	CI upper
Model 1A	QoL (CASP‐19)	Mood states associated with bipolar disorder	‐1.70	.21	<.001	−2.11	−1.28
Model 1B	QoL (CASP‐19)	Mood states associated with bipolar disorder	−1.37	.20	<.001	−1.78	−.97
		Loneliness	−.79	.02	<.001	−.82	−.75
		Social isolation	−.21	.02	<.001	−.25	−.17
		Education level					
		Intermediate	.62	.06	<.001	.49	.75
		Higher education	1.10	.08	<.001	.94	1.25
		Alcohol use					
		Irregular drinker	.43	.09	<.001	.25	.62
		Regular drinker	.74	.09	<.001	.56	.92
		Economic status					
		Quartile 2	.58	.76	<.001	.43	.72
		Quartile 3	.79	.78	<.001	.63	.93
		Quartile 4	.97	.81	<.001	.81	1.13
		Age	.01	.02	<.001	.01	.02
		Sex (female)	.47	.04	<.001	.39	.55
		Wave number					
		2	.06	.06	.356	−.07	.18
		3	.06	.06	.374	−.07	.18
		4	−.03	.06	.694	−.15	.09
		5	−.25	.07	<.001	−.38	−.13
		6	−.38	.07	<.001	−.52	−.24
		7	−.39	.07	<.001	−.52	−.25

*Note*: Model 1A: (*n* = 54,565 observations, 14,819 participants, 567 with mood states associated with bipolar disorder). Model 1B: (*n* = 47,149 observations, 13,265 participants, 485 with mood states associated with bipolar disorder).

Abbreviation: *B*, beta; BS SE, bootstrapped standard error; CI, confidence interval; DV, dependent variable; IV, independent variable; QoL, quality of life.

### Predictors of quality of life among older adults with mood states associated with bipolar disorder

#### Model 2

For Model 2, the authors ran a separate adjusted model that only included participants who reported experiencing mood states associated with bipolar disorder with the above covariates used to examine what predicts poorer quality of life in this group. Increased loneliness was found to significantly predict poorer quality of life (*B*: −1.25; SE .17; CI −1.57 to −.92; *p* < .001). Having completed intermediate (*B*: 1.30; SE .17; CI .10–2.51; *p* = .033) or higher education compared to no education (*B*: 1.89; SE .70; CI .52–3.25; *p* = .007), higher economic status (quartile 3, *B*: 2.09; SE .67; CI .78–3.40; *p* = .002 and quartile 4, *B*: 1.80; SE .77; CI .30–3.31; *p* = .019), and being female (*B*: 1.02; SE .40; CI .24–1.80; *p* = .011) significantly predicted better quality of life in this group. Older age also significantly predicted better quality of life in older adults with mood states associated with bipolar disorder, although the effect was relatively weak (*B*: .09; SE .03; CI .02–.15; *p* = .008; Table 3).

**TABLE 3:** Predictors of quality of life in older adults with mood states associated with bipolar disorder.

Model	DV	IV	*B*	BS SE	*p*	CI lower	CI upper
Model 2 (mood states associated with bipolar disorder only)	QoL (CASP‐19)	Loneliness	−1.25	.17	<.001	−1.57	−.92
		Social isolation	−.33	.22	.879	−.46	.39
		Education level					
		Intermediate	1.30	.61	.033	.10	2.51
		Higher education	1.89	.70	.007	.52	3.25
		Alcohol use					
		Irregular drinker	.38	.74	.607	−1.07	1.83
		Regular drinker	1.41	.88	.108	−.31	3.12
		Economic status					
		Quartile 2	1.41	.77	.065	−.09	2.92
		Quartile 3	2.09	.67	.002	.78	3.40
		Quartile 4	1.80	.77	.019	.30	3.31
		Age	.09	.03	.008	.02	.15
		Sex (female)	1.02	.40	.011	.24	1.80
		Wave number					
		2	−.80	.76	.296	−2.30	.70
		3	−.34	.73	.639	−1.76	1.08
		4	−.66	.79	.405	−2.21	.90
		5	−.79	.78	.319	−2.32	.80
		6	−.34	1.41	.807	−3.11	2.42
		7	−2.04	1.54	.184	−5.05	.97

*Note*: Model 2: (*n* = 711 observations, 485 with mood states associated with bipolar disorder).

Abbreviations: *B*, beta; BS SE, bootstrapped standard error; CI, confidence interval; DV, dependent variable; IV, independent variable; QoL, quality of life.

A sensitivity analysis was completed to address concerns surrounding the conceptual overlap between the CASP‐19 measure and independent variables included in the analysis (loneliness and social isolation). To complete the sensitivity analysis, items C4 (‘I feel left out of things’) and P4 (‘I enjoy being in the company of others’) were removed from the CASP‐19 item. Models 1B and 2 were then retested using the adapted CASP‐17 measure (see Data S1). The results highlighted that the findings were broadly consistent with the original analysis and that the effect sizes appeared to be slightly stronger in the sensitivity analysis where the new reduced CASP variable was included (see Data S1).

## DISCUSSION

The primary objective of this study was to test the hypothesis that experiencing mood states associated with bipolar disorder would predict poorer quality of life in older adults. A secondary aim was to assess which factors predicted quality of life in participants experiencing mood states associated with bipolar disorder. In support of the primary hypothesis, mood states associated with bipolar disorder significantly predicted poorer quality of life, even when controlling for multiple covariates (age, sex, social isolation, loneliness, economic status, education level, alcohol use). This effect was relatively small; older adults indicating that they experienced mood states associated with bipolar disorder scored 1.7 points lower on the CASP‐19 compared to older adults without mood states associated with bipolar disorder. In response to Hypothesis 2, loneliness significantly predicted poorer quality of life in older adults with mood states associated with bipolar disorder. Being female and having a high level of education predicted better quality of life in this group. Older age also had a very weak effect but was a statistically significant predictor of better quality of life in older adults with mood states associated with bipolar disorder.

As far as the authors are aware, this is the first study to examine an epidemiological sample to demonstrate that mood states associated with bipolar disorder significantly predict poorer quality of life in older adults whilst controlling for multiple relevant covariates. Our findings appear consistent with the work of Depp et al. (2006), who reported that older adults with bipolar disorder experienced poorer wellbeing than healthy controls. This adds evidence that the poorer quality of life observed in younger age groups with bipolar disorder (Michalak et al., 2006) persists into later life and requires attention.

As observed in older adults in the general population (Ong et al., 2016) and younger adults with bipolar disorder (Giacco, 2023), loneliness appears to be problematic for older adults experiencing mood states associated with bipolar disorder. Loneliness is defined as a lack of desired companionship by Bekhet et al. (2008). It is not possible to determine the causes of loneliness in the current analysis, although it is plausible that factors such as lower perceived social support among older adults with mood states associated with bipolar disorder could contribute to this finding (Beyer et al., 2003). Mood states associated with bipolar disorder are also thought to cause significant disruption to relationships and are linked to higher rates of divorce, which may leave people experiencing increased loneliness in later life (Granek et al., 2016). In contrast, social isolation was not found to be a predictor of poorer quality of life in older adults with mood states associated with bipolar disorder. Social isolation is a related but distinct concept to loneliness, defined as the objective lack of social contact with the community, family, and friends (Fakoya et al., 2020). The literature investigating social isolation among older adults with bipolar disorder is limited. However, the finding that social isolation does not predict poorer quality of life in this study could potentially be linked to Blixen et al. (2016) paper reporting that individuals with bipolar disorder often attempt to cope with challenges by socializing with others. This may limit social isolation in this group, although further research is needed to examine the causes and consequences of loneliness and social isolation among older adults with mood states associated with bipolar disorder.

Higher educational attainment was positively associated with better quality of life in individuals with mood states associated with bipolar disorder. People with bipolar disorder have been found to commonly experience oppression and stigma that creates barriers to them achieving higher educational attainment (Kruse & Oswal, 2018). Consequently, attempting to remove these barriers may be an important intervention that enables individuals with bipolar disorder to maintain improved wellbeing. Consistent with other studies investigating alcohol consumption and older age, regular drinking was associated with improved quality of life for older adults with mood states associated with bipolar disorder in this study. The effects of alcohol use on quality of life among older adults have been attributed to its associations with increased social support, stress relief, and improved networking (Chan et al., 2009). Being female also significantly predicted increased quality of life in older adults with mood states associated with bipolar disorder. These findings are opposed to the limited available literature examining sex differences in bipolar disorder. Existing research suggests that females with bipolar disorder experience poorer functioning due to issues such as severe postpartum episodes, increased obesity, and greater risk of rapid cycling (Baskaran et al., 2014; Blanken et al., 2024; Diflorio & Jones, 2010; Erol et al., 2015). It is unclear why there are discrepancies between the findings of this study and the existing literature that investigate gender differences in bipolar disorder in younger and older adults (Baskaran et al., 2014; Blanken et al., 2024; Diflorio & Jones, 2010; Erol et al., 2015). However, the broad categorization of mood states associated with bipolar disorder in this study may have resulted in some differences in these findings. Although participants in this study reported experiences of psychiatric mood swings that appeared to be associated with bipolar disorder, it is possible that the experiences of participants in the ELSA sample may not be representative of older adults formally diagnosed with bipolar disorder. Finally, older age was found to significantly predict increased quality of life in older adults experiencing mood states associated with bipolar disorder, although the effect was very weak. At present, literature examining the experiences of the ‘oldest old’ with bipolar disorder (aged 75 and over; Gwozdz & Sousa‐Poza, 2010) is sparse, potentially due to the increased mortality and reduced lifespan associated with this diagnosis (Sajatovic et al., 2015). Further research that explores sex differences and the experiences of the ‘oldest old’ may identify important clinical implications in these groups.

### Clinical implications

The current study illuminates the consequences of ageing whilst experiencing mood states associated with bipolar disorder and suggests that this group require increased support to improve their quality of life. Despite this, literature surrounding older adults with bipolar disorder is insufficient; there is inadequate focus on how to adapt care to meet the needs of this population (Dols et al., 2016). Services should be aware that older adults with bipolar disorder potentially experience different challenges to younger age groups with bipolar disorder, meaning individualized, person‐centred care is required (Ljubic et al., 2021). In particular, loneliness was found to be a highly significant predictor of poorer quality of life in this group and interventions should attempt to address this to improve outcomes.

### Strengths and limitations

There are several limitations to this research. First, although ELSA is a large epidemiological sample, the number of individuals reporting mood states was relatively small, but sufficient to complete multilevel modelling. Second, the analysis was cross‐sectional meaning it was not possible to draw inferences around causality. Third, we did not control for some potentially key covariates (e.g., substance misuse, medication use, history of psychological therapy) as the data was not available within ELSA. Finally, due to low response rates for people with bipolar disorder, we combined this variable with those who experienced psychiatric mood swings. It is possible that this variable may not be representative of the experiences of people with bipolar disorder and participants' self‐reported experiences of mood in this study. However, this decision to combine these variables was driven by the available data and consultation with patient and public advisors and allowed for greater statistical power in the analysis.

### Future research directions

Future research should aim to build upon the findings of this study and attempt to identify the causal factors associated with bipolar disorder that contribute to poorer quality of life through large‐scale longitudinal research. In the future, it would be useful for analysis to explore the complex interplay between variables related to mood states associated with bipolar disorder and quality of life whilst exploring differences between subgroups within population‐based data. Additionally, exploring the relationship between ageing with mood states and loneliness may help to illuminate why this appeared to be problematic in the current study. Improving our knowledge of the causes and predictors of poor quality of life could help us to better understand the key care needs of older adults with mood states associated with bipolar disorder, highlight necessary adaptations to care, enhance support, and potentially improve the quality of life for this population moving forward. Finally, investigating differences in quality of life between younger and older adults with bipolar disorder may help to identify potential interaction effects between ageing and bipolar disorder.

## CONCLUSION

In conclusion, this analysis suggests that mood states associated with bipolar disorder significantly predict poorer quality of life in older adults, supporting our initial hypothesis. Loneliness is a predictor of poorer quality of life in older adults experiencing mood states associated with bipolar disorder. Being female and having higher educational attainment may support older adults with mood states to maintain better quality of life as they age. However, the specific factors that contribute to these findings require further investigation. Improving our understanding of the mechanisms that lead to poorer quality of life among older adults with mood states associated with bipolar disorder may enable us to provide more tailored, appropriate, and effective support to this group. At present, older adults with mood states associated with bipolar disorder appear to experience significant challenges that require increased attention within clinical services to support this group to age well and improve their wellbeing in later life.

## AUTHOR CONTRIBUTIONS

**Aaron Warner:** Conceptualization; writing – original draft; writing – review and editing; formal analysis; methodology. **Carol Holland:** Conceptualization; methodology; supervision; writing – review and editing. **Fiona Lobban:** Conceptualization; methodology; writing – review and editing; supervision. **Lee Bentley:** Formal analysis; writing – review and editing; conceptualization; methodology; supervision. **Elizabeth Tyler:** Conceptualization; writing – review and editing; methodology; formal analysis; supervision. **Jasper Palmier‐Claus:** Conceptualization; writing – review and editing; methodology; formal analysis; supervision.

## FUNDING INFORMATION

This report is independent research funded by the National Institute for Health Research Applied Research Collaboration North West Coast (ARC NWC). The views expressed in this publication are those of the author(s) and not necessarily those of the National Institute for Health Research or the Department of Health and Social Care.

## CONFLICT OF INTEREST STATEMENT

None.

## Supporting information


Data S1.

## Data Availability

The data that support the findings of this study are available from the corresponding author upon reasonable request.
