# Supplementary material for: Quality of life in older adults with mood states associated with bipolar disorder: A secondary analysis of the English longitudinal study of ageing data
Source: Br J Clin Psychol. 2024 Aug 11;64(2):188–202. doi: 10.1111/bjc.12495 (PMC12057322; doi:10.1111/bjc.12495)
Supplement: Supplementary file 1 — Data S1. [file BJC-64-188-s001.docx]

**Quality of life in older adults with mood states associated with bipolar disorder: A secondary analysis of The English Longitudinal Study of Ageing data**

**Supplementary Materials (sensitivity analysis)**

**Model 1B (CASP-17)**

| **Model** | **DV** | **IV** | ***B*** | **BS SE** | ***P*** | **CI lower** | **CI upper** |  |
| --- | --- | --- | --- | --- | --- | --- | --- | --- |
|  |  |  |  |  |  |  |  |  |
|  |  |  |  |  |  |  |  |  |
| **Model 1B** | **QoL (CASP-17)** | **Mood states associated with bipolar disorder** | -2.17 | 0.26 | <0.001 | -2.68 | -1.65 |  |
|  |  | **Loneliness** | -1.85 | 0.02 | <0.001 | -1.90 | -0.80 |  |
|  |  | **Social isolation** | -0.16 | 0.02 | <0.001 | -0.21 | -0.11 |  |
|  |  | **Education level** |  |  |  |  |  |  |
|  |  | Intermediate | 0.65 | 0.09 | <0.001 | 0.48 | 0.83 |  |
|  |  | Higher education | 1.16 | 0.11 | <0.001 | 0.93 | 1.38 |  |
|  |  | **Alcohol use** |  |  |  |  |  |  |
|  |  | Irregular drinker | 0.94 | 0.12 | <0.001 | 0.71 | 1.16 |  |
|  |  | Regular drinker | 1.78 | 0.12 | <0.001 | 1.53 | 2.01 |  |
|  |  | **Economic status**  Quartile 2  Quartile 3  Quartile 4 | 1.50  2.24  2.98 | 0.10  0.10  0.10 | <0.001  <0.001  <0.001 | 1.30  2.04  2.77 | 1.70  2.45  3.19 |  |
|  |  | **Age** | -0.06 | 0.03 | <0.001 | -0.07 | -0.06 |  |
|  |  | **Sex (female)** | 1.02 | 0.05 | <0.001 | 0.92 | 1.12 |  |
|  |  | **Wave number** |  |  |  |  |  |  |
|  |  | 2 | -0.19 | 0.07 | 0.010 | -0.34 | 0.04 |  |
|  |  | 3 | -0.09 | 0.07 | 0.232 | -0.24 | 0.06 |  |
|  |  | 4 | -0.43 | 0.08 | <0.001 | -0.56 | -0.28 |  |
|  |  | 5 | 0.17 | 0.08 | 0.030 | 0.02 | 0.33 |  |
|  |  | 6 | 0.10 | 0.09 | 0.231 | -0.07 | 0.27 |  |
|  |  | 7 | 0.08 | 0.08 | 0.349 | -0.09 | 0.25 |  |

**Model 2 – Mood states associated with bipolar disorder only (CASP-17)**

| **Model** | **DV** | **IV** | ***B*** | **BS SE** | ***P*** | **CI lower** | **CI upper** |
| --- | --- | --- | --- | --- | --- | --- | --- |
| **Model 2 (Mood states associated with bipolar disorder only)** | **QoL (CASP-17)** | **Loneliness** | -2.39 | 0.22 | <0.001 | -2.81 | -1.96 |
|  |  | **Social isolation** | -0.06 | 0.34 | 0.853 | -0.60 | 0.73 |
|  |  | **Education level**  Intermediate  Higher education | 0.90  3.44 | 0.58  0.88 | 0.120  <0.001 | -0.24  1.71 | 2.04  5.17 |
|  |  | **Alcohol use**  Irregular drinker  Regular drinker | 1.54  2.66 | 0.99  1.11 | 0.122  0.016 | -0.41  0.49 | 3.51  4.84 |
|  |  | **Economic status**  Quartile 2  Quartile 3  Quartile 4 | 2.74  5.06  6.47 | 1.12  1.05  1.14 | 0.015  <0.001  <0.001 | 0.54  2.99  2.99 | 4.93  7.12  8.70 |
|  |  | **Age** | 0.04 | 0.04 | 0.314 | -0.04 | 0.12 |
|  |  | **Sex (female)** | 1.86 | 0.50 | <0.001 | 0.88 | 2.84 |
|  |  | **Wave number**  2  3  4  5  6  7 | -0.15  0.42  -0.88  -0.91  -1.01  -2.07 | 0.92  0.99  0.94  1.02  1.64  1.99 | 0.875  0.672  0.351  0.373  0.539  0.298 | -1.95  -1.51  -2.72  -2.92  -4.22  -5.96 | 1.66  2.36  0.96  1.09  2.20  1.83 |
